# Surgical Extirpation of Apical Left Ventricular Thrombus in Takotsubo Cardiomyopathy

**DOI:** 10.1155/2015/387037

**Published:** 2015-05-26

**Authors:** Tetsuya Niino, Satoshi Unosawa

**Affiliations:** Department of Cardiovascular Surgery, National Hospital Organization Disaster Medical Center, Japan

## Abstract

We report a patient with takotsubo cardiomyopathy who underwent surgical resection of apical left ventricular thrombus. A 59-year-old woman was transferred to our hospital in shock with hypothermia and diabetic ketoacidosis. The electrocardiogram showed ST segment elevation, while echocardiography revealed a reduced ejection fraction with apical and midventricular akinesis. Emergency coronary angiography showed normal coronary arteries, so takotsubo cardiomyopathy was diagnosed. Follow-up echocardiography revealed improvement of the ejection fraction. A mobile apical thrombus was also detected. Thrombectomy was performed via a left apical incision and postoperative recovery was uneventful.

## 1. Introduction

Takotsubo cardiomyopathy, which is also known as transient left ventricular apical ballooning syndrome, typically occurs in persons under severe emotional or physical stress. Takotsubo cardiomyopathy induced by diabetic ketoacidosis has also been reported [1].

The prognosis of takotsubo cardiomyopathy is generally favorable, with normalization of ventricular wall motion occurring after a number of weeks, but serious complications such as heart failure, mural thrombus, thromboembolism, ventricular tachycardia, and cardiac rupture have been reported [2, 3].

We present a case of takotsubo cardiomyopathy complicated by left ventricular apical thrombus, in which the thrombus was successfully removed via apical left ventriculotomy.

## 2. Case Report

**Figure 1:**
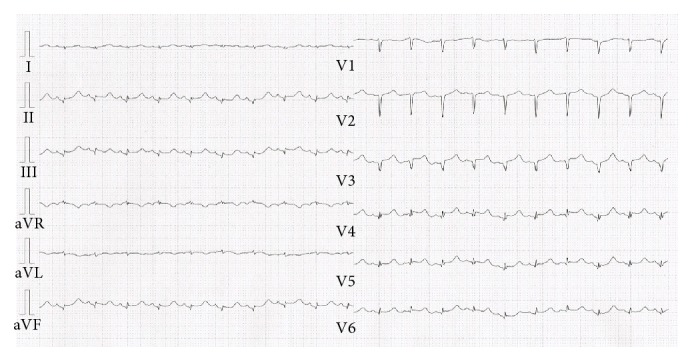
Electrocardiogram showed significant ST-elevation in leads II, III, aV_F_, and V_3–6_.

A 59-year-old woman was admitted to our institution with unconsciousness. She had no relevant medical history. On admission, she had shock state and systolic blood pressure was 63 mmHg, with dehydration and prominent Kussmaul breathing. Her temperature was 30.9°C and her blood glucose was 1018 mg/dL. Blood gas analysis revealed severe metabolic acidosis (pH: 6.89) and an increased base excess (−30.4 mmol/L). Electrocardiography showed significant ST segment elevation in leads II, III, aV_F_, and V_3–6_ (Figure 1).

The patient remained in shock despite treatment of her hypothermia and dehydration. Transthoracic echocardiography (TTE) demonstrated apical and midventricular akinesis of the left ventricle, with preservation of basal contraction. The ejection fraction (EF) was reduced to less than 25%. Cardiac enzymes were elevated, with creatine kinase being 1951 IU/L, creatine kinase-MB being 230 IU/L, and troponin I being 7.94 ng/mL. Coronary angiography was performed immediately. Because her coronary arteries were normal, takotsubo cardiomyopathy was diagnosed. The patient was treated with an intra-aortic balloon pump (IABP) for hemodynamic support and anticoagulation with heparin. On the 2nd day, she was weaned from the IABP. TTE showed improvement of the EF to 40–50% with no apical thrombus, so heparin was stopped on day 6. New-onset type 1 diabetes mellitus was also diagnosed and she started insulin therapy.

**Figure 2:**
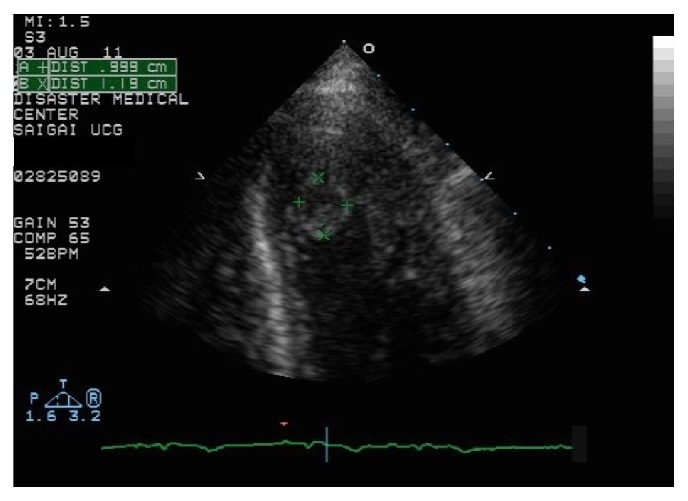
Echocardiogram showed a mobile mural thrombus in the apex of left ventricle.

On day 13, TTE revealed normalization of left ventricular wall motion but also showed a mobile round thrombus at the apex of the ventricle (Figure 2). Anticoagulant therapy with warfarin was started, and we explained the risks and benefits of surgical treatment and anticoagulant therapy. The patient has agreed on surgical thrombectomy, which was indicated because of the embolic potential in a patient recovering from takotsubo cardiomyopathy.

Median sternotomy was performed and cardiopulmonary bypass was established from the superior and inferior vena cava to the ascending aorta. After cross-clamping the ascending aorta, cold crystalloid cardioplegic solution was administered antegradely via the aortic root. An incision was made at the apex of the left ventricle parallel to the left anterior descending artery and the thrombus was extirpated. The thrombus was fragile and less than 1 cm in size. Then the ventriculotomy was closed with a buttressed mattress structure and a continuous suture. The aortic cross-clamping time, cardiopulmonary bypass time, and operating time were 29, 67, and 139 minutes, respectively. The present patient was weaned from cardiopulmonary bypass using low-dose dopamine (2 *µ*g/kg/min), but this was unnecessary at end of surgery.

Pathological examination of apical myocardial tissue revealed interstitial fibrosis and rupture of myocardial fibers, which were findings suggestive of myocarditis. Her postoperative course was uneventful and postoperative echocardiography revealed normal left ventricular function.

## 3. Discussion

The prognosis of patients with takotsubo cardiomyopathy is generally favorable, although death sometimes occurs due to complications such as heart failure, mural thrombus, thromboembolism, or cardiac rupture [2, 3].

De Gregorio et al. reported intracavitary thrombus in 2.5% of patients with takotsubo cardiomyopathy and embolic complications in 33.3% of those with left ventricular thrombosis, including stroke, renal infarction, and popliteal thrombosis [4]. Accordingly, anticoagulant therapy is recommended for patients with takotsubo cardiomyopathy, but thromboembolism has been reported in some patients despite anticoagulation with heparin or warfarin [5]. It has been reported that plasma catecholamine levels are markedly elevated in patients with takotsubo cardiomyopathy, suggesting the possibility of catecholamine-induced platelet activation and aggregation [6–8].

However, takotsubo cardiomyopathy complicated ventricular apical rupture due to transmural myocardial necrosis with hemorrhage has been reported. Therefore, whether anticoagulant therapy with warfarin should be routinely performed in these patients to prevent thrombosis remains controversial.

Surgery and anesthesia have been associated with takotsubo cardiomyopathy, and it has been suggested that excessive circulating levels of catecholamines due to surgical stress may induce this cardiomyopathy [9]. Dobutamine stress test has also possibility of induced takotsubo cardiomyopathy [10].

The thromboembolism occurred in 0 to 20% with left ventricular thrombus in takotsubo cardiomyopathy under anticoagulation therapy [11–13]. Management of left ventricular thrombus in patients with takotsubo cardiomyopathy is controversial; conservative therapy has the possibility to be effective and safe if the thrombi are smooth, conform to the cavity shape, and are relatively stable. But the risk of embolism is relatively high when the thrombus is located in the apical region and surgical thrombectomy needs to be considered, especially in patients with a mobile thrombus [14]. There have been previous reports about thrombectomy via apical left ventriculotomy or a transmitral approach, although the latter gives a poor view of the target [13, 15]. In our patient, apical ventriculotomy provided good access for thrombectomy.

In conclusion, we found that left apical ventriculotomy was useful to approach an apical thrombus, and surgical thrombectomy is an option for left apical thrombus in patients with takotsubo cardiomyopathy.
